# Synthetic Tuning of Co^II^-Doped Silica Nanoarchitecture Towards Electrochemical Sensing Ability

**DOI:** 10.3390/nano10071338

**Published:** 2020-07-09

**Authors:** Olga Bochkova, Mikhail Khrizanforov, Aidar Gubaidullin, Tatiana Gerasimova, Irek Nizameev, Kirill Kholin, Artem Laskin, Yulia Budnikova, Oleg Sinyashin, Asiya Mustafina

**Affiliations:** 1 Arbuzov Institute of Organic and Physical Chemistry, FRC Kazan Scientific Center, Russian Academy of Sciences, Arbuzov str., 8, 420088 Kazan, Russia; khrizanforov@gmail.com (M.K.); aidar@iopc.ru (A.G.); tatyanagr@gmail.com (T.G.); irek.rash@gmail.com (I.N.); kholin06@mail.ru (K.K.); olefindirector@gmail.com (Y.B.); oleg@iopc.ru (O.S.); asiyamust@mail.ru (A.M.); 2 Kazan Federal University, Kremlevskaya str. 29/1, 420008 Kazan, Russia; artemka166@mail.ru

**Keywords:** silica nanoparticles, cobalt (II) dopant, spectral properties, nanoarchitecture, organophosphorous compounds, electrochemical sensing

## Abstract

The present work introduces both synthesis of silica nanoparticles doped with Co^II^ ions by means of differently modified microemulsion water-in-oil (w/o) and Stöber techniques and characterization of the hybrid nanoparticles (Co^II^@SiO_2_) by TEM, DLS, XRD, ICP-EOS, SAXS, UV-Vis, and UV-Vis/DR spectroscopy and electrochemical methods. The results reveal the lack of nanocrystalline dopants inside the hybrid nanoparticles, as well as no ligands, when Co^II^ ions are added to the synthetic mixtures as Co^II^(bpy)_3_ complexes, thus pointing to coordination of Co^II^ ions with Si-O^-^ groups as main driving force of the doping. The UV-Vis/DR spectra of Co^II^@SiO_2_ in the range of d-d transitions indicate that Stöber synthesis in greater extent than the w/o one stabilizes tetrahedral Co^II^ ions versus the octahedral ions. Both cobalt content and homogeneity of the Co^II^ distribution within Co^II^@SiO_2_ are greatly influenced by the synthetic technique. The electrochemical behavior of Co^II^@SiO_2_ is manifested by one oxidation and two reduction steps, which provide the basis for electrochemical response on glyphosate and HP(O)(OEt)_2_ with the LOD = 0.1 μM and the linearity within 0.1–80 μM. The Stöber Co^II^@SiO_2_ are able to discriminate glyphosate from HP(O)(OEt)_2_, while the w/o nanoparticles are more efficient but nonselective sensors on the toxicants.

## 1. Introduction

Silica nanoparticles provide a good platform for uploading of metal ions and complexes through different synthetic approaches, including doping into silica matrix or deposition at a surface of nanoparticles [1,2,3,4,5,6,7,8,9,10,11,12,13,14,15,16]. Such hybrid silica nanoparticles uploaded by transition d-metal ions exhibit electrochemical behavior controlled by both inner-sphere environments of the metal ions and nano-architecture of the nanoparticles [14,15,17,18]. Controllable electrochemical behavior is a prerequisite for an application of the hybrid nanoparticles in electrochemical catalysis or sensing. In turn, nanoheterogeneous catalysts and sensors are of particular importance due to their greater stability and reusability versus the molecular complexes [14,15,17,18]. The substrate dependent electrochemical behavior of transition metal ions and complexes is the basis for electrochemical detection of many pollutants, including organophosphorous ones [19,20,21,22,23,24,25,26,27,28,29]. Electrochemical methods have many advantages over other sensing techniques such as reproducibility, good stability, high sensitivity, measuring trace level of samples and cost-effectiveness [19,30,31]. This requires an engineering of new materials as components of electrodes with advanced electrochemical functions [32,33,34]. Both unique redox activity and easy encapsulation into polymeric materials make hybrid silica nanoparticles rather promising components of the electrodes for electrochemical sensing [35], although metal oxide or metal-based nanomaterials have got wider application in the engineering of the devices for electrochemical sensing [36,37]. Thus, design and synthesis of the hybrid nanoparticles with controllable inner-sphere environment of d-metal ions and their distribution within the nanoparticles is a rather challenging task from both fundamental and practical points of view.

However, successful doping or deposition of d-metal complexes without significant changes in the ligand environment can be performed only for kinetically inert complexes, which are exemplified in literature by tris-dipyridyles of Co^III^ and Ru^II^ [16,38,39,40]. The labile d-metal complexes, including those of Ni^II^ and Co^II^, can also be doped into silica nanoparticles, although their ligand environment scarcely remains unchanged after the doping [14,15]. Moreover, literature data introduce both works reporting on safe doping of the Co^II^ complexes [2,9] and those revealing complex formation of Co^II^ ions with Si–O^−^-groups as the reason for the ligand substitution [41]. The mutual impacts of electrostatic attraction of the complexes or coordinative bonding of Co^II^ ions with Si-O^−^-groups to the doping procedure can be recognized by comparative structural analysis of the Co^II^-dopants inside silica spheres. Thus, both Co^II^(bpy)_3_ (bpy is 2,2′-bipyridine) and CoCl_2_ will be used as the precursors for the synthesis of Co^II^-doped silica nanoparticles.

The water-in-oil microemulsion (w/o) and Stöber procedures are most widely applied for doping of hydrophilic dopants such as d-metal salts or complexes into silica spheres [14,15,16,38,39,40]. As it was previously reported, the choice of w/o or Stöber procedure for doping of metal ions into silica nanoparticles has a great impact on both content of the dopants and their electrochemical and catalytic activity [14,15,16]. Thus, the present work is aimed at highlighting a role of the synthetic procedure and nature of the precursor on the structure of the Co^II^-dopants and nanoarchitecture of Co^II^-doped silica nanoparticles. In turn, the structural features of Co^II^-dopants and Co^II^-doped silica nanoparticles are correlated with their electrochemical behavior and sensing ability in order to reveal an applicability of the differently synthesized Co^II^-doped silica nanoparticles for the sensing purposes. The water-in-oil microemulsion and Stöber procedures were chosen as the synthetic methods with the use of both Co^II^(bpy)_3_ (bpy is 2,2′-bipyridine) and CoCl_2_ as the precursors. The structural features of the hybrid silica nanoparticles were analyzed on both molecular and nano-levels by dynamic light and small angle X-ray scattering, electronic microscopy, elemental analysis and UV-Vis, UV-Vis DR, and IR spectroscopy methods. Electrochemical behavior of the hybrid nanoparticles was revealed in order to evaluate their applicability for the sensing. The sensing ability of the hybrid nanoparticles was exemplified by two organophosphorous compounds (OPC), which are the well-known pollutant glyphosate [42] and diethyl phosphite. The latter was chosen as a model of decomposition products of OPC, which are often no less toxic than the precursors.

## 2. Materials and Methods

### 2.1. Materials

Tetraethyl orthosilicate (TEOS, 98%), ammonium hydroxide (28–30%), n-heptanol (98%), cyclohexane (99%), 2.2′-bipyridine (bpy, 99%), diethyl phosphite (98%) were obtained from Acros Organics (Geel, Belgium); Triton X-100, cobalt(II) chloride (97%), cobalt(II) tetrafluoroborate hexahydrate (99%), N-(Phosphonomethyl)glycine (Glyphosate) were purchased from Sigma-Aldrich (St. Louis, Missouri, USA). CH_3_CN (CHROMASOLV^®^ Plus, ≥99.9%, by Acros Organics, Geel, Belgium) was used without any preliminary purification. The Ethanol, Acetone, and TEOS were purified by distillation.

[Co(bpy)_3_](BF_4_)_2_ was synthesized according to the known procedure [43]. To an ethanol solution of Co(BF_4_)_2_·6H_2_O (2 × 10^−2^ mol·L^−1^) (100 mL), an ethanol solution (50 mL) of bpy ligand (6 × 10^−2^ mol·L^−1^) was added under stirring. The resulting solution was stirred for 1 h, then filtered and left to evaporate at room temperature. After several days, air-stable, brown crystals of this complex were obtained. Yield: 83%. The elemental analysis data for C_30_H_24_B_2_CoF_8_N_6_ are presented in Table 1. The spectroscopic data for [Co(BF_4_)_2_(bpy)_3_] matched that reported in the literature [43].

Et_4_NBF_4_ was obtained by mixing an aqueous solution of Et_4_NOH (30–35%) with HBF_4_ to a neutral indicator reaction. Et_4_NBF_4_ precipitated from the reaction mixture as white crystals, which were separated by filtering. The powder salt was further recrystallized from diethyl ether and dried for 2 to 3 days in a vacuum at 55 °C for dehydration.

#### Silica Nanoparticles Preparation

Water-in-oil (w/o) microemulsion procedure [44] was the following. A mixture of Triton X-100 (2.38 g), n-heptanol (2.29 mL), cyclohexane (9.32 mL), TEOS (0.2 mL), and 1.1 mL of bidistilled water (for “empty” silica nanoparticles) or aqueous solution (1.1 mL) of the Co^II^-precursor (Co(bpy)_3_(BF_4_)_2_ or CoCl_2_, C = 4.5 × 10^−3^ mol·L^−1^) was prepared and stirred for 30 min. The obtained w/o microemulsion was mixed with a microemulsion containing Triton X-100 (2.38 g), n-heptanol (2.29 mL), cyclohexane (9.32 mL) and aqueous solutions of NH_3_ (28–30%) with stirring. After 24 h of stirring, both Co^II^-doped and empty silica nanoparticles were precipitated from the microemulsion by adding acetone with further centrifugation. The nanoparticle precipitate was washed by solutions of ethanol-acetone (1:1), ethanol (two times), and water (several times) to remove any residual surfactant molecules and organic solvents. Physically adsorbed Co^II^ ions or complexes and Triton X-100 molecules at nanoparticles surface were removed by ultrasonication during the washing procedure. Pink-colored Co^II^-doped and white “empty” silica nanoparticles were obtained.

*Stöber synthesis* [14] was done through the following steps. A solution of TEOS (1.14 mL) in EtOH (11.36 mL) was added to NH_4_OH (28–30%) (0.38 mL) in EtOH (8.05 mL)-H_2_O (4.05 mL) at the speed 2 mL per min (thought syringe pump) under continuous stirring (750 rpm). After 6 min, the solution of [Co(bpy)_3_](BF_4_)_2_ or CoCl_2_ in EtOH (2.5 mL, C = 4.5 × 10^−3^ mol·L^−1^) was injected into this mixture. In the case of the “empty” silica nanoparticles, the solution of TEOS (1.14 mL) in EtOH (11.36 mL) was added to NH_4_OH (28–30%) (0.38 mL) in EtOH (10.55 mL)-H_2_O (4.05 mL) at the speed 2 mL per min (thought syringe pump) under continuous stirring (750 rpm). After 6 h of stirring, silica nanoparticles were precipitated by centrifuging and washing several times by ethanol and water. To remove physically absorbed Co^II^ complexes or ions from the particles surfaces, an ultrasonication was used during the washing procedure. Blue-colored Co^II^-doped and white “empty” silica nanoparticles were obtained.

The electrochemical, PXRD, SAXS, IR, and Raman experiments were performed with dried samples of the silica nanoparticles.

### 2.2. Samples Characterization

Transmission electron microscopy (TEM) measurements were carried out on Hitachi HT7700 transmission electron microscope (Chiyoda-ku, Japan) with energy-dispersive X-ray detector from Thermo Scientific. The accelerating voltage was equal to 80 kV. The accumulation time of one spectrum was 300 s.

Co and Si were identified in the silica nanoparticles colloids using a simultaneous inductively coupled plasma atomic emission spectrometry (ICP-AES) model iCAP 6300 DUO by Varian Thermo Scientific Company equipped with a CID detector. This spectrometer enables the simultaneous measurement of peak heights within the 166 to 867 nm range. The optical resolution is less than 0.007 nm to 200 nm. As for the working frequency, it is 27.12 MHz. Together, the radial and axial view configurations enable optimal peak height measurements with suppressed spectral noises.

UV-Vis spectra of solutions and silica nanoparticles dispersions were recorded on Specord^R^50 Plus (Analytikjena, Germany). SNs dispersions were ultrasonicated within 10 min before using.

Powdered samples were characterized by UV-Vis/DR technique using a Jasco V-650 spectrophotometer (Jasco International Co. Ltd., Hachioji, Tokyo, Japan) equipped with an integrating sphere accessory for diffuse reflectance spectra acquisition. BaSO_4_ powder was used as the reference for baseline correction.

The dynamic light scattering (DLS) measurements were performed by means of the Malvern Mastersize 2000 particle analyzer (Malvern, UK). A He-Ne laser operating at 633 nm wavelength and emitting vertically polarized light was used as a light source. The measured autocorrelation functions were analyzed by Malvern DTS software and the second-order cumulant expansion methods. The effective hydrodynamic radius (RH) was calculated by the Einstein–Stokes relation from the first cumulant: D = kBT/6phRH, where D is the diffusion coefficient, kB is the Boltzmann constant, T is the absolute temperature, and h is the viscosity. The diffusion coefficient was measured at least three times for each sample. The average error in these experiments was approximately 4%. All samples were prepared from the bidistilled water with prior filtering through the PVDF membrane using the Syringe Filter (0.45 µm). Zeta potential “Nano-ZS” (MALVERN) using laser Doppler velocimetry and phase analysis light scattering was used for zeta potential measurements. Silica nanoparticles dispersions were ultrasonicated within 10 min before using.

IR spectra of solid samples have been registered using a Bruker Vector-27 FTIR spectrometer in the 400–4000 cm^−1^ range (optical resolution 4 cm^−1^). The samples were prepared as KBr pellets.

Raman spectra were registered at room temperature using a BRUKER RAM II module attached to a BRUKER VERTEX 70 FTIR spectrometer (excitation 1064 nm, Ge detector at liquid nitrogen temperature, back-scattering configuration; range 10–4000 cm^−1^, optical resolution 4 cm^−1^, scan number 1024).

Electrochemical measurements were taken on a BASiEpsilonE2P electrochemical analyzer (West Lafayette, IN, USA). The program concerned Epsilon-ECUSB-V200 waves. A conventional three-electrode system was used with glassy carbon for carbon paste electrode (CPE) solutions for powder samples as the working electrode, the An Fc/Fc system serving as reference electrode, and a Pt wire as the counter electrode. In addition, 0.1 M [Et_4_N][BF_4_] was used as the supporting electrolyte to determine the current voltage characteristics. The solvent for all measurements was water. To study the powder samples, a modified CPE working electrode was used, which was prepared as follows: the carbon particles/phosphonium salt (dodecyl(tri-tert-butyl)phosphonium tetrafluoroborate) composite electrode was prepared using a grinding a mixture of graphite powder and phosphonium salt with a 90/10 (w/w) ratio in mortar giving it a homogeneous mass [45]. A modified electrode was also devised in a similar manner except that a portion (ca. 5%) of the graphite powder was replaced by the CPs under study. As a result, a portion of the resulting paste was packed firmly into the (3 mm in diameter) Teflon holder cavity.

Powder X-ray diffraction (PXRD) measurements were performed on a Bruker D8 Advance diffractometer equipped with Vario attachment and Vantec linear PSD, using Cu radiation (40 kV, 40 mA) monochromated by the curved Johansson monochromator (λ Cu K_α1_ 1.5406 Å). Room-temperature data were collected in the reflection mode with a flat-plate sample. The samples were loaded on a standard zero diffraction silicon plate, which was kept spinning (15 rpm) throughout the data collection. Patterns were recorded in the 2*Θ* range between 3° and 93°, in 0.008° steps, with a step time of 0.1–4.0 s. Several diffraction patterns in various experimental modes were collected and summed for the sample. Processing of the obtained data performed using EVA software packages [46].

Small angle X-Ray scattering (SAXS) data for samples were collected with the Bruker AXS Nanostar system using CuKα (λ 1.5418Å) radiation from a 2.2 kW X-ray tube (40 kV, 35 mA) coupled with Gobbel mirrors optics and a HiStar 2D area detector. The beam was collimated using three pinholes with apertures of 800, 450, and 700 μm. The instrument was operated with a sample-to-detector distance of 63.5 cm to provide data at angles 0.1°< 2θ< 4.8°, which correspond to 0.007 Å−1 < s < 0.34 Å−1. The value of s is proportional to the inverse of the length scale (s = (4π/λ)sin(θ) in units of Å^−1^). Scattering patterns were obtained for the samples at 23 °C in an evacuated chamber. The measurements were performed in transmission mode with the use of glass capillaries filled by powder samples. The capillaries (2 mm diameter) were sealed and put into evacuated chamber by means of the holders. For each sample, several experiments were performed, allowing for controlling the quality of the experiments. The results of the experiments are summarized, so that the total time of each experiment was equal to 30,000 s. The 2D scattering patterns were integrated using the SAXS program package [47]. Calculation of structural parameters, simulation, and graphical representation of the results were performed using a PRIMUS [48] program package.

## 3. Results and Discussion

### 3.1. Size, Cobalt Content, and Spectral Properties of the Hybrid Nanoparticles (CoII@SiO2) in Correlation with the Synthetic Conditions

**Figure 1:**
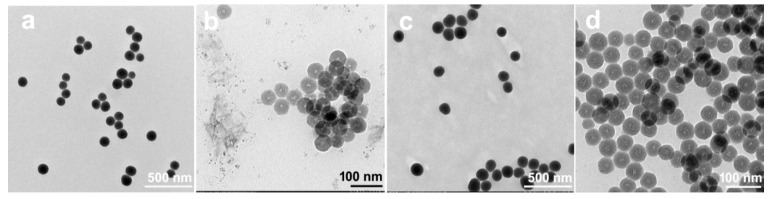
TEM-images of Co^II^@SiO_2_ nanoparticles obtained by: Stober procedure (**a**,**c**) and water-in-oil microemulsion technique (**b**,**d**) on the basis of Co^II^(bpy)_3_ (**a**,**b**) and CoCl_2_ (**c**,**d**) as dopants.

**Scheme 1:**
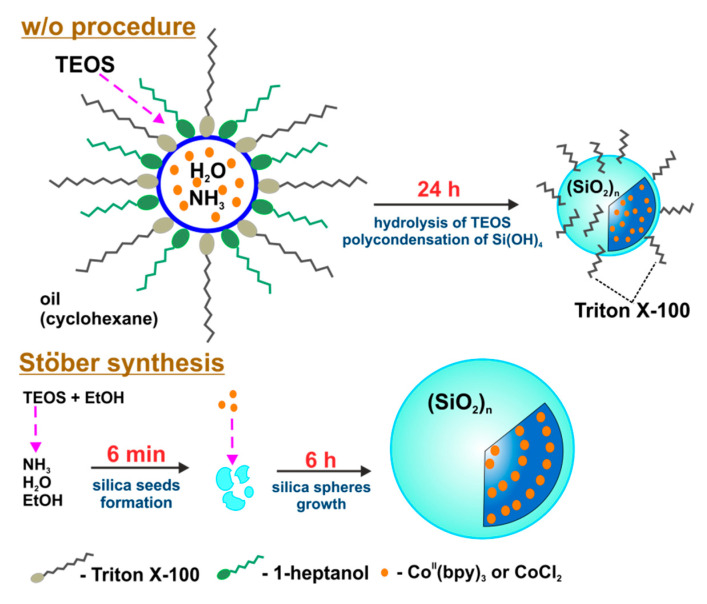
Schematic representation of different techniques for synthesis of Co^II^@SiO_2_.

Stöber and water-in-oil microemulsion (w/o) techniques provide a facile approach for encapsulation of charged metal complexes into silica nanoparticles [2,9,14,15,16,38,39,40]. The use of the both techniques results in the synthesis of the hybrid silica nanoparticles. Figure 1 illustrates the TEM images of the hybrid nanoparticles, while their diameters evaluated from the images and the Si:Co ratios calculated from the ICP-EOS data are collected in Table 2. The data in Table 1 indicate that the size and the content of Co^II^ evaluated by the Si:Co ratios are to a major extent dependent on the synthetic technique, while smaller differences derive from the doping of Co^II^(bpy)_3_ or CoCl_2_. The difference in the synthetic methods is illustrated by Scheme 1. The size of the nanoparticles synthesized by the w/o technique is controlled by the TX-100-based reverse micelles (Scheme 1). Thus, water:oil volume ratios along with surfactant and co-surfactant concentrations have a great impact on the size control in the framework of the w/o technique. The sizes of silica nanoparticles grown by the Stöber method are mainly affected by concentrations of TEOS and ammonia, while the ethanol:water volume ratio plays a smaller role in controlling the size (Scheme 1). Moreover, the water soluble Co^II^-dopants are concentrated within nano-droplets of the reverse micelles, which explains the greater uploading by Co^II^ ions for the nanoparticles synthesized by the w/o procedure in comparison with those obtained by the Stöber method (Table 2, Scheme 1). It is also worth noting that the color of both aqueous colloids and dried samples of the hybrid nanoparticles are pink when they are synthesized by the w/o, while the Stöber procedure results in the blue nanoparticles. Similar to the above-mentioned factors the color of the hybrid nanoparticles seems to be independent from their doping with Co^II^(bpy)_3_ or CoCl_2_.

**Figure 2:**
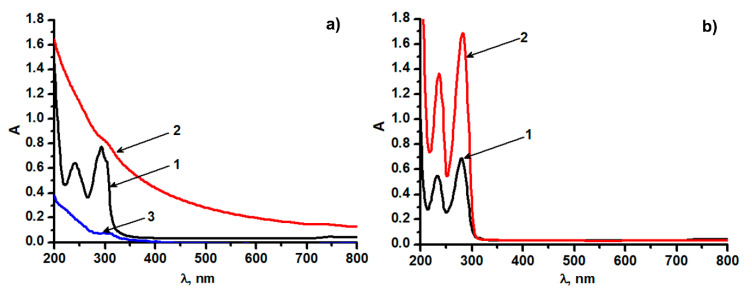
UV-Vis absorbance spectra in ethanol of: (**a**) 1—Co(bpy)_3_(BF_4_)_2_ (C = 1.67 × 10^−5^ mol·L^−1^), Co^II^@SiO_2_ (C = 0.2 g·L^−1^) on the basis of Co^II^(bpy)_3_ obtained by different techniques: 2—Stöber, 3—w/o; (**b**) 1—bpy (C = 5 × 10^−5^ mol·L^−1^), 2—supernatant after Stöber synthesis diluted 10 times.

The presence of bpy ligands both coordinated with Co(II) and as “free” molecules inside silica nanoparticles can be revealed by UV-Vis spectroscopy measurements in both supernatants (Figure 2b) and aqueous dispersions of the synthesized silica nanoparticles (Figure 2a). The analysis of the UV-Vis spectral data reveals the lack of the bands peculiar for Co^II^(bpy)_3_ in the aqueous colloids for both types of Co^II^@SiO_2_ (curves 2 and 3 in Figure 2a). The supernatant after the synthesis by the Stöber procedure reveals the release of bpy from the complex (Figure 2b), while the electronic absorption bands of Triton X-100 mask the presence of bpy in the supernatant after the synthesis by the w/o method (Figure S1). Moreover, its concentration in the supernatant indicates the degradation of about 57% of Co^II^(bpy)_3_ added to the synthetic mixture.

**Figure 3:**
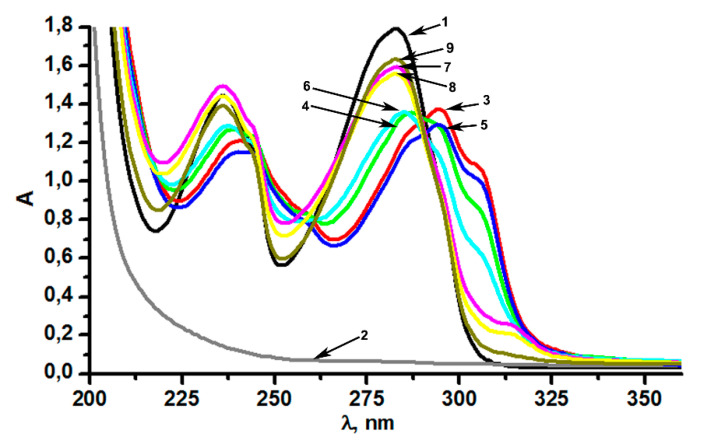
UV-Vis absorbance spectra in ethanol of: 1—bpy (C = 5 × 10^−5^ mol·L^−1^); 2—TEOS; 3—Co(bpy)_3_(BF_4_)_2_ (C = 4 × 10^−5^ mol·L^−1^); Co^II^(bpy)_3_ (C = 4 × 10^−5^ mol·L^−1^) in the presence of: 4—ammonia hydroxide, 5—TEOS, 6—NH_4_OH and TEOS, 7—NH_4_OH after 40 min of stirring, 8—NH_4_OH and TEOS after 40 min of stirring (Co:TEOS:NH_3_ ratio was the same as in the Stöber synthetic conditions); 9—Co^II^(bpy)_3_ after addition in the synthetic mixture according to synthesis condition (Stöber synthesis in Materials and Methods section) and this mixture was diluted 10 times.

**Figure 4:**
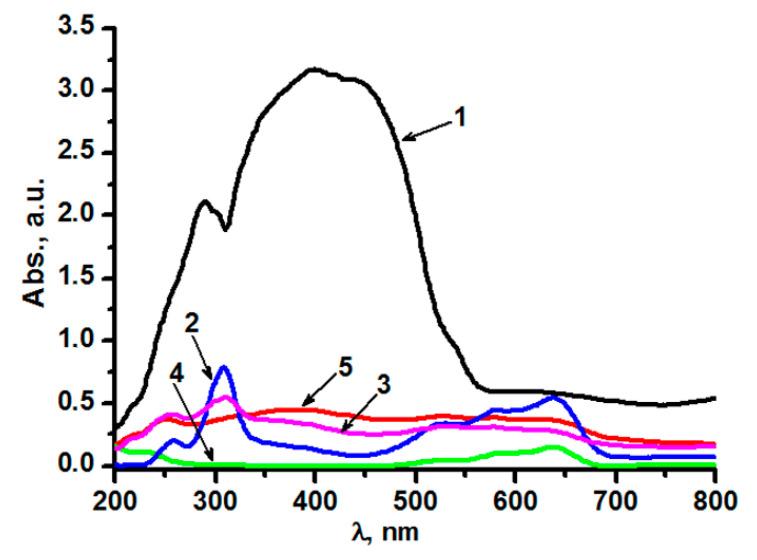
UV–Vis diffuse reflectance spectra of: Co^II^(bpy)_3_ (1); and Co^II^@SiO_2_ synthesized by different techniques: Stöber with Co^II^(bpy)_3_ (2) and CoCl_2_ (4); w/o with Co^II^(bpy)_3_ (3) and CoCl_2_ (5).

Thus, the color changes during the synthesis of the nanoparticles by the w/o and Stöber procedures derive from the transformation of the spectral pattern from Co^II^(bpy)_3_ to that of bpy (Figure 3). In particular, the yellow color seems to remain unchanged in aqueous or alcohol solution of Co(bpy)_3_(BF_4_)_2_ exactly after addition of tetraethoxysilane and ammonia (4–8 in Figure 3), which is confirmed by the small changes in the spectral pattern of Co^II^(bpy)_3_. The color changes followed by the spectral transformations from that of Co^II^(bpy)_3_ to that of bpy (3 and 1 in Figure 3) is observed within 6 or 30 min after beginning of the Stöber (9 in Figure 3) or w/o synthesis. The time durations for 6 and 30 min correlate with the appearance of silica seeds in the framework of Stöber [14,39] and w/o [49] procedures. It is worth assuming that the growth of silica seeds with Si-O^-^ groups exposed at their surface provides a reason for the transformations of Co^II^(bpy)_3_ to bpy and Co^II^ ions due to a complex formation of the latter with Si-O^-^ groups of the silica seeds. The assumption can be confirmed by comparison of the UV-Vis diffuse reflectance spectra in the long-wave spectral range derived from the d-d transitions of the hybrid nanoparticles doped with Co(dipy)_3_(BF_4_)_2_ or CoCl_2_ (Figure 4). For these purposes, the UV-Vis/DR spectra of Co^II^@SiO_2_ were recorded for their dried samples along with the powder of Co(dipy)_3_(BF_4_)_2_ (Figure 4) and “empty” silica spheres (SiO_2_) (Figure S2) for the comparison. The latter were synthesized by the same w/o and Stöber procedures without the addition of the Co^II^-dopants. The characterization of SiO_2_ synthesized in the same synthetic conditions as the Co^II^@SiO_2_ by TEM indicate that their sizes are greatly affected by the synthetic method (48 ± 5 and 97 ± 5 nm for SiO_2_ synthesized by w/o and Stöber methods correspondingly), while the effect of the dopants is rather small (compare with the sizes in Table 2).

The spectra in Figure 4 indicate the difference in the spectral pattern within 500–700 nm for the Co^II^@SiO_2_ synthesized by the w/o and Stöber procedures, while the nature of the Co^II^-precursor (Co(bpy)_3_(BF_4_)_2_ or CoCl_2_) insignificantly affects the d-d transitions of the Co^II^@SiO_2_. The spectra of Co^(II)^@SiO_2_ synthesized with the use of Co^II^(bpy)_3_ as precursor (2 and 3 in Figure 4) differ from the one of Co^II^(bpy)_3_ complex in the range associated with d-d transitions, but demonstrate the band at ~300 nm, absent in the spectra of Co^(II)^@SiO_2_ with CoCl_2_ (curve 4 and 5 in Figure 4), associated most probably with “free” bpy. The spectra of the Stöber Co^II^@SiO_2_ (curve 2 and 4 in Figure 4) demonstrate three strong bands at 528, 580, and 638 nm, which are characteristic for ^4^A_2_(F) → ^4^T_1_(P) transitions in tetrahedral Co^II^ chromophores [50,51,52,53]. It should be mentioned that the same spectral pattern (bands at 547, 585, 633 nm) has been observed for tetrahedral Co^II^ ion coordinated to four oxygen atoms in 1D coordination polymer of Co(fcdHp), based on 1,1′-ferrocenylenbis(H-phosphinic) acid (H2fcdHp) [54]. The similar bands in this region (curve 3 and 5 in Figure 4) are not so pronounced in the spectra of the w/o Co^II^@SiO_2_. They also demonstrate another rearrangement of intensities. Most probably, the spectral pattern of the w/o Co^II^@SiO_2_ samples derives from Co^II^ ions in octahedral environment, for which weak bands at ca. 500–580 nm assigned to ^4^T_1g_ → ^4^A_2g_(F) transitions are characteristic [50,53,55]. The absorbance intensity of tetrahedral Co^II^ is more intense (∼100 times) than that of octahedral Co^II^ due to the lack of an inversion center in the former symmetry [56]. Therefore, the contribution from the octahedral Co^II^ to the spectral pattern could be masked by the bands arisen from the tetrahedral Co^II^ chromophores. Nevertheless, both rearrangement of bands intensities at 500–700 nm and their lowering in spectra 3 and 5 compared to 2 and 4 (Figure 4) allows one to suggest the greater contribution of the octahedral Co^II^ chromophores in the w/o Co^II^@SiO_2_ versus the Stöber Co^II^@SiO_2_. It is noteworthy that the coordinative bonds between the Co^II^ ions and Si-O^-^ groups at the silica surface are the key reason for the formation of the Co^II^@SiO_2_ by the w/o and the Stöber procedures. However, it is worth assuming that the extents of tetrahedral and octahedral Co^2+^ chromophores are greatly affected by the aqueous or alcohol environment of Co^II^ ions in the frameworks of the w/o or Stöber techniques (Scheme 1).

**Figure 5:**
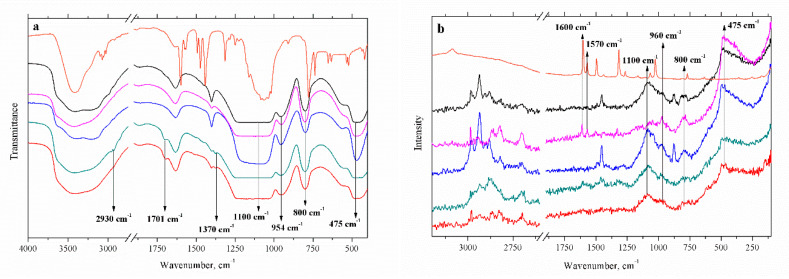
IR (**a**) and Raman (**b**) spectra of Co^II^(bpy)_3_ (orange), SiO_2_ (black) and Co^II^@SiO_2_ synthesized by the w/o on the basis of Co^II^(bpy)_3_ (cyan) and CoCl_2_ (red) and by Stöber on the basis of Co^II^(bpy)_3_ (magenta) and CoCl_2_ (blue).

The infrared and Raman spectra of Co^II^@SiO_2_ nanoparticles (Figure 5) synthesized by both methods are very similar and demonstrate characteristic for silica nanoparticles bands: 475 cm^−1^, attributed to bending modes between the Si–O–Si bonds, 800 cm^−1^, assigned to the symmetric stretching vibration between Si–O–Si bonds, ~960 cm^−1^ that is often linked with the stretching vibration of Si–OH bond and ~1100 cm^−1^, which corresponds to the asymmetric stretching vibration of Si–O–Si bond in the SiO_4_ tetrahedron as reported in literature [41,57,58,59,60]. The IR spectra of NPs obtained by the w/o technique (cyan and red in Figure 5a) contain additional bands at ~1370, 1701, and 2930 cm^−1^ most probably associated with CH bending and C=O and alkyl CH stretching vibrations of acetone and TX-100 molecules (cyan and red in Figure 5a). The presence of TX-100 molecules derives from their efficient adsorption at the surface of silica nanoparticles (Scheme 1) leading to the so-called surfactant-based corona similar with the previously reported F-127-based corona [61] of the silica nanoparticles synthesized by the w/o procedure with F-127 as the surfactant. Raman spectra of Stöber Co^II^@SiO_2_ based on Co^II^(bpy)_3_ contain very weak bands of bpy at ~1600 and 1570 cm^−1^ (magenta in Figure 5b), which are absent in the spectrum of “empty” SiO_2_ (black in Figure 5b), and are not visible in their IR spectra (magenta in Figure 5a).

### 3.2. Nanoarchitecture of Co^II^@SiO_2_ and SiO_2_ in Correlation with the Synthetic Procedure

**Figure 6:**
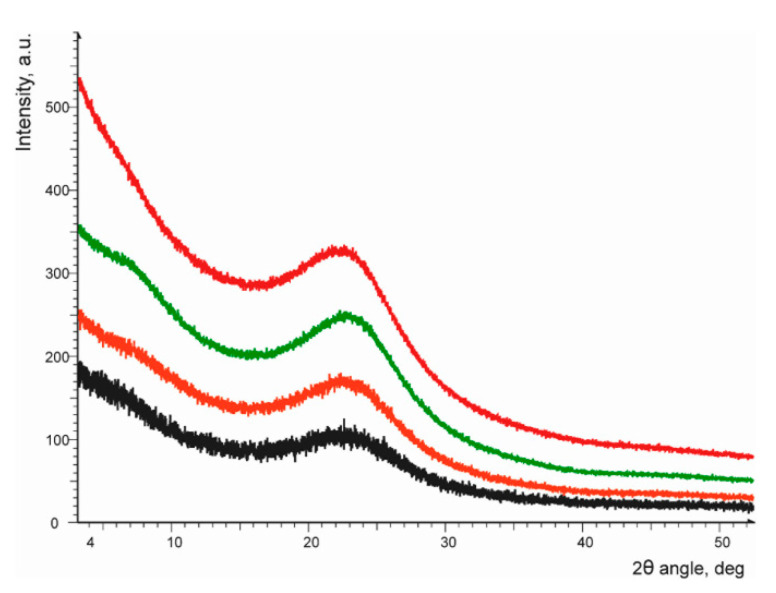
PXRD patterns: black—w/o SiO_2_, orange—Stöber SiO_2_, red—w/o Co^II^@SiO_2_, green—Stöber Co^II^@SiO_2_. The curves are shifted relative to each other along the intensity axis for clarity.

Electrochemical behavior of metal ions encapsulated into silica nanoparticles is affected by the inner-sphere environment of metal ions and their preferable localization within interior (core) or exterior (shell) zones of the hybrid nanoparticles [15,62]. Thus, nanoarchitecture of the hybrid nanoparticles manifested by different distribution of the dopant within their core and shell zones was studied by powder X-ray diffraction (PXRD) and small angle X-ray scattering (SAXS) techniques. Combination of the techniques was used to reveal both supramolecular and nano-structuring in the dried samples of Co^II^@SiO_2_ and SiO_2_ synthesized by the both methods. It is well-known that silica spheres synthesized by w/o or Stöber techniques are manifested by widened peaks in PXRD spectra, while the presence of nano-crystallites derived from the cobalt salts or oxides can be revealed by the appearance of the specific peaks [41,62,63]. Literature data indicate that the amorphous nature of silica remains practically unchanged under coordination of Co(II) ions with Si-O^-^ groups, while the narrow peaks revealing the nanocrystallites of cobalt oxide derive from the extra-amounts of the doped cobalt salt [41,62]. Such nanocrystallites can be revealed by comparative analysis of PXRD data of Co^II^@SiO_2_ nanoparticles synthesized by both methods and their empty analogous (SiO_2_). The PXRD spectra recorded for the dried samples of Co^II^@SiO_2_ and SiO_2_ nanoparticles synthesized by the both methods (Figure 6) demonstrate the PXRD patterns peculiar for amorphous silica nanoparticles, which agrees well with the concentration conditions in the synthesis of the Co^II^@SiO_2_ (see the Exp. Section for more details). However, the weak widened peaks in the range of small angles revealed in the PXRD curves of Stöber Co^II^@SiO_2_ and SiO_2_ derive from the inclusions of paracrystalline phase with short- and medium-range ordering into the amorphous nanoparticles.

SAXS has been previously documented as successful technique to reveal nano-structuring deriving from silica nanoparticles, as well as inhomogeneity in electron density distribution within nanoparticles [41,64,65,66]. It is worth noting in this connection that the sizes of Co^II^@SiO_2_ and SiO_2_ nanoparticles synthesized by Stöber method (Table 2 and Table 3) lie above the values available for correct evaluation by the method (1–60 nm), while the sizes of smaller Co^II^@SiO_2_ and SiO_2_ nanoparticles synthesized by the w/o procedure can be evaluated from the SAXS measurements. However, the X-ray scattering profiles of the Co^II^@SiO_2_ and SiO_2_ (Figure S3) reveal their nanostructuring manifested by the presence of nano-aggregates with the sizes below 60 nm even for the Stöber Co^II^@SiO_2_ and SiO_2_. The nanostructuring arisen from inhomogeneity of electron density distribution within the silica nanoparticles may be quantitatively described in a “core-shell” framework. However, the shell zones of such “core-shell” nanoparticles along with the interparticle space can be considered as isotropic homogeneous medium with the electron density lower than that of the core zone. It is worth assuming that the core zones of the Co^II^@SiO_2_ and SiO_2_ as nanoparticles encapsulated into an isotropic medium with distances between their centers above the sizes of the core zones. Thus, the quantitative analysis of the SAXS data was performed in the assumption that the scattering results from noninteracting spherical nanoparticles uniformly enclosed in a homogeneous medium which provides their local monodisperse environment. Two-dimensional scattering curves were obtained by averaging of the eight X-ray scattering measurements for both hybrid and empty silica nanoparticles, while no effect of the prolonged X-ray irradiation on the scattering data was revealed (Figure S4).

Table 3 collects the calculated from the SAXS data parameters characterizing nanoparticles, such as radius of gyration (R_g_ and R_g_*) evaluated by the Guinier method and from analysis of distance distribution function respectively, biggest distances in the particles (D_max_), and average diameter of the particles in a sphere-shaped model framework (d_s_, d_s_ = √(5/3) × R_g_ × 2). The latter can be compared with the d-values evaluated by TEM, which are also collected in Table 2 to facilitate the comparison. This difference is too great to be explained by the difference in the techniques. The d_s_ values calculated from SAXS are at the level of 33–38 nm for Co^II^@SiO_2_ and SiO_2_ nanoparticles synthesized by the w/o technique; these values are lower than the d values evaluated by TEM on about 10 nm (Table 3). In the framework of the aforesaid assumption, the sizes revealed by the SAXS measurements refer to the core zones of the w/o Co^II^@SiO_2_ and SiO_2_. This doesn’t exclude the presence of an exterior layer exhibiting smaller electronic density than the silica spheres. The above-mentioned TX-100-corona at the surface of w/o Co^II^@SiO_2_ and SiO_2_ is one of the reasons for an exterior layer with the electron density smaller than that arisen from the silica matrix. Therefore, the deviation between the d_s_ and d values can be explained by the fact that the sizes revealed by the TEM images derive from both core and shell zones of the w/o Co^II^@SiO_2_ and SiO_2_. It is also worth noting that the D_max_ values exceed the d_s_ ones on no more than 5 nm (Table 3), which, in turn, indicates that the shape of the w/o Co^II^@SiO_2_ and SiO_2_ revealed by SAXS deviates from an ideal spherical shape.

The evaluated from the SAXS data fractal dimensions (FD) of w/o Co^II^@SiO_2_ and SiO_2_ presented in Table 3 are above 3.0. Such FD values are peculiar for surficial fractality derived from the folded surface, whose density is lower than that of the silica matrix. This is in good agreement with the aforesaid assumption about the TX-100-based corona at the silica surface as schematically shown in Scheme 1. Comparative analysis of both FD and R_s_ values reveals the insignificant difference between “empty” SiO_2_ and Co^II^@SiO_2_ w/o nanoparticles (Table 3). This similarity indicates that the distribution of the Co^II^ ions within Co^II^@SiO_2_ (w/o) is rather homogeneous without preferable localization within specific zones of the nanoparticles, while the lower electronic density of the exterior layer derives from the specificity of the w/o technique (Scheme 1).

The SAXS analysis of Stöber Co^II^@SiO_2_ and SiO_2_ is the peculiar case, since the greatest R_g_ values refer to the d_s_-values at the level of 34–41 nm which are far below the d-values evaluated from the TEM images (Table 3, Figure S5). Moreover, more smooth profiles of the scattering curves measured for Stöber Co^II^@SiO_2_ and SiO_2_ in comparison with the w/o ones (Figure S3) indicate greater polydispersity of the Stöber versus the w/o nanoparticles. This deviation points to inhomogeneity in electron density distribution within Stöber Co^II^@SiO_2_ and SiO_2_, which agrees well with the zones of short- and medium-range ordering revealed from the PXRD curves of the corresponding nanoparticles (Figure 6). The shapes of the revealed core zones also deviate from ideal spheres, which is evident from the deviation between D_max_ и d_s_ values (Table 3). Moreover, the FD value of the empty silica spheres made by Stöber procedure is on the level of surficial fractality (3.37), while the FD values of the Stöber Co^II^@SiO_2_ (2.6–2.7) refer to the so-called mass fractality which indicate the inhomogeneous distribution of Co^II^ ions within the Stöber Co^II^@SiO_2_. This is quite different from the homogeneous distribution within the w/o Co^II^@SiO_2_, where both FD and d_s_ values of the “empty” SiO_2_ and Co^II^@SiO_2_ nanoparticles are at the same level.

In summary, the SAXS data revealed that the synthesis of Co^II^@SiO_2_ in the framework of Stöber method results in the localization of Co^II^-dopant within specific zones of the silica spheres, while more homogeneous distribution of Co^II^-dopant within Co^II^@SiO_2_ is obtained by the w/o technique. Moreover, the surface fractality of Co^II^@SiO_2_ (w/o) can be explained by the porous silica surface decorated by TX-100-based corona.

### 3.3. Electrochemical Behavior of Co^II^@SiO_2_

**Figure 7:**
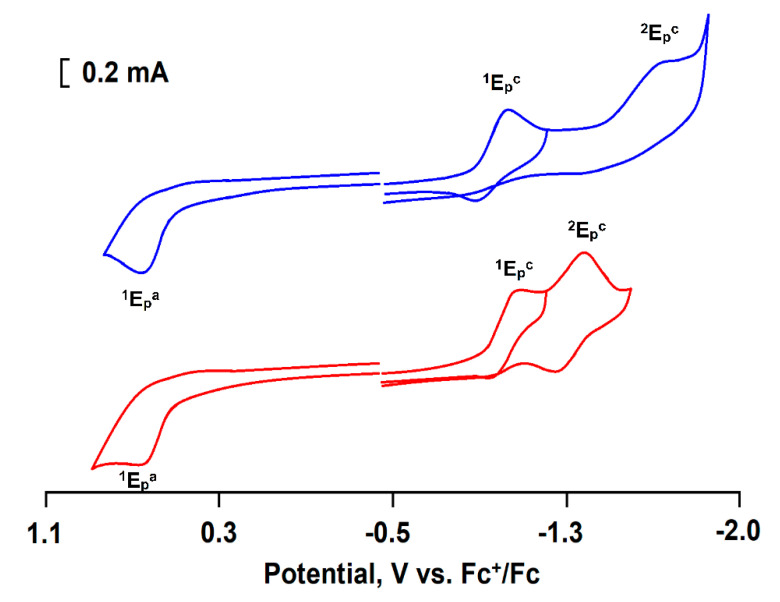
CVs for w/o Co^II^@SiO_2_ (red) and Stöber Co^II^@SiO_2_ (blue) WE: CPE, H_2_O, 10^−1^ mol·L^−1^ Et_4_NBF_4_ Potentials vs. Ag/AgCl recalculated to Fc^+^/Fc.

The electrochemical behavior of Co^II^@SiO_2_ was measured with the use of carbon-paste electrode (CPE) [45]. The CPE-based technique enables avoiding effects of different colloid stability, which makes it most convenient for comparative analysis of electrochemical behavior of Co^II^ encapsulated into Co^II^@SiO_2_ synthesized by the Stöber and w/o methods. Two types of Co^II^@SiO_2_, i.e., (Stöber) and (w/o) have identical first oxidation and reduction peaks. In particular, one-electron irreversible oxidation of Co^II^ to Co^III^ is observed at a potential of 0.65 V (Figure 7). At −1.12–−1.15 V, a one-electron reversible reduction of Co^II^ to Co^I^ is observed for both types of nanoparticles, while a significant difference is observed at the second reduction of Co^I^ to Co^0^ peak. The irreversible reduction of Co^I^ to Co^0^ recorded at −1.69 V is revealed for Stöber Co^II^@SiO_2_, while, for w/o Co^II^@SiO_2_, the reduction at −1.38 V is reversible (Figure 7, Table 4).

It is well-known that reduction or oxidation potentials are to a great extent dependent on the inner-sphere environment of Co^II^. However, the previous report reveals that predominant localization of the Ni^II^ complexes within core zone of the Ni^II^-doped silica nanoparticles shifts the electrochemical Ni^II^ to Ni^I^ reduction to more negative potentials [15]. Thus, the second Co^I^ to Co^0^ reduction step is affected, while the Co^II^ to Co^I^ reduction and Co^II^ to Co^III^ oxidation potentials are not influenced by the synthetic method (Table 4). The results are not enough to recognize the exact reasons for the aforesaid similarities and differences in electrochemical behavior of Stöber Co^II^@SiO_2_ and w/o Co^II^@SiO_2_. Nevertheless, the electrochemical activity of the Co^II^@SiO_2_ points to their applicability in electrochemical analysis of OPC.

### 3.4. Co^II^@SiO_2_ for Electrochemical Determination of OPC

The presentation of an electrochemical response of Co^II^@SiO_2_ on OPC is worth preceding by the discussion of main mechanisms responsible for the response. Both oxidation and reduction of Co^II^ centers in Co^II^@SiO_2_ are manifested in the CVs by the peaks with the peak currents dependent on concentration of the Co^II^ centers. The increased peak current of Co^II^/Co^III^ or Co^II^/Co^I^ transformations results from regeneration of Co^II^ centers due to rapid interaction of Co^III^ or Co^I^ with OPC. In turn, the peak current can be significantly decreased in a case of unfavorable processes (the destruction of the catalytically active form or complex formation preventing a reestablishing of Co^II^ centers).

**Figure 8:**
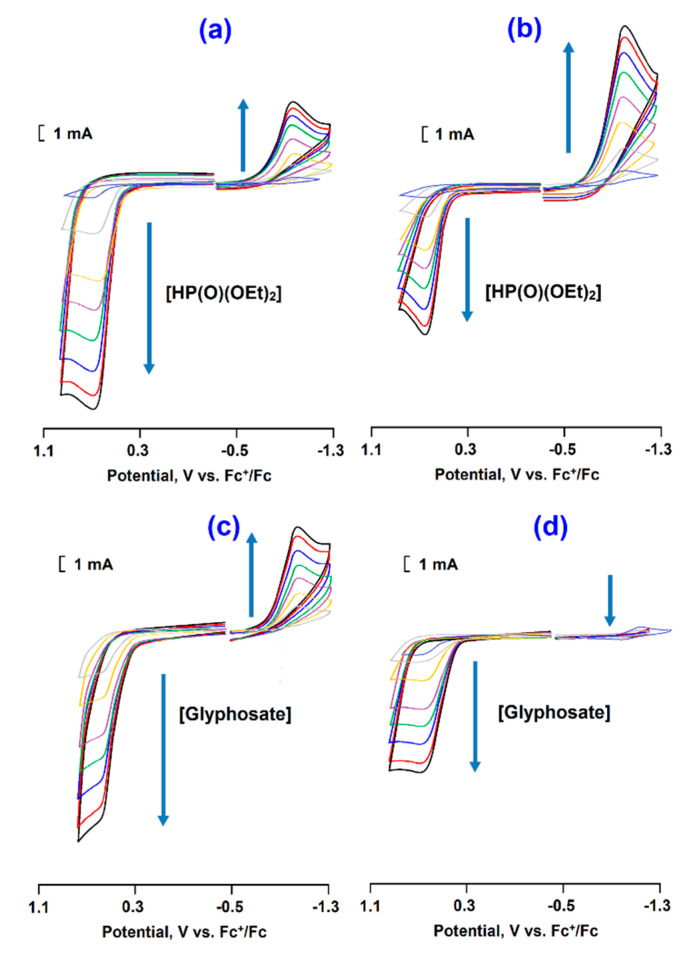
CVs for w/o Co^II^@SiO_2_ (**a**,**c**) and Stöber Co^II^@SiO_2_ (**b**,**d**) in the absence and in the presence of increasing quantities of HP(O)(OEt)_2_ (**a**,**b**) and Glyphosate (**c**,**d**) (1.0, 2.5, 5.0, 10.0, 25.0, 50.0 mmol·L^−1^). WE: CPE, H_2_O, 10^−1^ mol·L^−1^ Et_4_NBF_4_ Potentials vs. Ag/AgCl recalculated to Fc^+^/Fc. Arrows indicate the direction of current changes.

Both OPC (glyphosate and diethyl phosphite) are electrochemically inactive in the studied interval of potentials. Thus, the enhanced peak current at the oxidation and reduction potentials of w/o Co^II^@SiO_2_ under the increased concentrations of the OPC (Figure 8a,c) indicates that oxidized Co^III^@SiO_2_ or reduced Co^I^@SiO_2_ forms return to the initial form (Co^II^@SiO_2_) due to rapid redox reaction of Co^III^ or Co^I^ centers with the OPC. The concentration dependent increase in the peak current (Figure 8a,c) provides the electrochemical response on the OPC. The comparative analysis of the substrate-induced electrochemical response of Co^II^@SiO_2_ synthesized by w/o and Stöber procedures reveal a significant difference between them. The difference is the highest at the reduction potential, when Stöber Co^II^@SiO_2_ nanoparticles demonstrate a significant increase in the peak current under the growth of diethylphosphite concentration (Figure 8c), while glyphosate in the same conditions drops the peak current to zero (Figure 8d). It is worth assuming that the aforesaid fact correlates with the differences in the inner- and outer-sphere of Co^II^ ions within the silica spheres formed by the w/o and Stöber methods. Thus, the OPC-triggered electrochemical behavior of Co^II^@SiO_2_ is also greatly influenced by the method (Stöber or w/o) applied for their synthesis. Unfortunately, exact mechanisms responsible for the different electrochemical behavior of the hybrid nanoparticles in the presence of the OPC are not clear from the results. Additional studies lying beyond the present work scope are required for their clarification.

**Figure 9:**
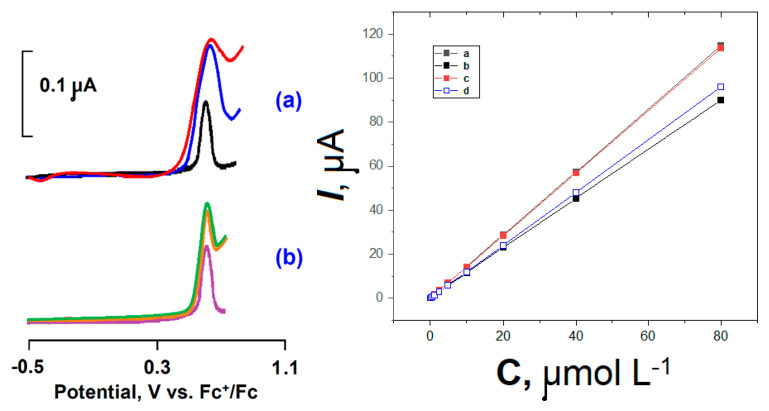
DPV voltammograms (**on the left**) of the Co^II^@SiO_2_ oxidation at CPE without and in the presence of increasing amounts of of phosphorus compounds (at the first addition of OPC). (**a**): w/o Co^II^@SiO_2_ (**black**); w/o Co^II^@SiO_2_ + 0.1 µmol·L^−1^ HP(O)(OEt)_2_ (**blue**); w/o Co^II^@SiO_2_ + 0.1 µmol·L^−1^ Glyphosate (**red**); (**b**): Stöber Co^II^@SiO_2_ (**purpure**); Stöber Co^II^@SiO_2_+ 0.1 µmol·L^−1^ HP(O)(OEt)_2_ (**orange**); Stöber Co^II^@SiO_2_+ 0.1 µmol·L^−1^ Glyphosate (**Green**); Potentials vs. Fc+/Fc. H_2_O, 10^−1^ mol·L^−1^ Et_4_NBF_4_. Corresponding plots of *i*_cat_ vs. OPC concentration (from 0.1 to 80 µmol·L^−1^: 0.1, 0.2, 0.3, 0.6, 1.2, 2.4, 5.0, 10.0, 20.0, 40.0, 80.0 µmol·L^−1^), **the inset on the right**: w/o Co^II^@SiO_2_ in the presence of Glyphosate (**a, gray**) and HP(O)(OEt)_2_ (**c, red**); Stöber Co^II^@SiO_2_ in the presence of Glyphosate (**d, blue**) and HP(O)(OEt)_2_ (**b, black**).

Nevertheless, the presented results clearly demonstrate the applicability of the substrate-induced electrochemical response for quantitative analysis of the OPC. The increase in the peak current within anodic potentials range is greater than that within the cathodic range. For evaluation of a lower detection level (LOD), the peak current of Co^II^@SiO_2_ at different concentrations of the OPC was analyzed by differential impulse voltammetry technique (Figure 9 and Figure S6). The results (Figure 9a,b) indicate that even small amounts of the OPC (0.1 μmol·L^−1^) induce the detectable peak increase; thus, this concentration level can be considered as the LOD-value. Moreover, the inset in Figure 9 indicates the linear relationship between the response currents and HP(O)(OEt)_2_ (red-black) and Glyphosate (gray-blue) concentrations in the range from 0.1 to 80 μM (with a correlation coefficient of 0.99). The slope of the linear dependencies is somewhat greater for w/o Co^II^@SiO_2_ versus Stöber Co^II^@SiO_2_, although the discrimination between glyphosate and diethylphosphite is rather poor for both types of Co^II^@SiO_2_. It is worth noting that glyphosate can be discriminated from diethylphosphite by the selective peak current increase in the cathodic range when the CPE was modified by Stöber Co^II^@SiO_2_.

The estimated LOD = 0.1 µmol·L^−1^ is greater than the values reported for most sensitive sensing systems [21], but lower than the values reported for the colorimetric sensor [67]. The linearity of the peak current increase is manifested in a wider concentration range than the linearity range reported in [21] for electrochemical sensors based on Cu-BTC/g-C_3_N_4_ nanosheets (g-C3N4 = graphite phase nitrogenized carbon, BTC = benzene-1,3,5-tricarboxylic acid). Moreover, the present LOD-value is enough for evaluation of dose limit of glyphosate in a drinking water. Furthermore, the Co^II^@SiO_2_ based sensors have the characteristics of short detection time, high stability and easy operation, which is of great impact on potential applications in electrochemical OPC analysis.

## 4. Conclusions

In summary, analysis of size and content of silica nanoparticles doped by Co^II^ ions by means of water-in-oil microemulsion (w/o) or Stöber synthetic technique confirms the applicability of the both techniques for synthesis of the hybrid nanoparticles. Monitoring of the transformations of [Co(bpy)_3_](BF_4_)_2_ used as the precursor for the hybrid nanoparticles through the UV-Vis spectral analysis during the synthesis highlights the decomposition of the precursor due to efficient coordination of the Co^II^ ions via Si-O^-^ groups exposed at the silica seeds. Thus, the use of either [Co(bpy)_3_](BF_4_)_2_ or CoCl_2_ as the precursors results in the nanoparticles with the same inner-sphere environment of the Co^II^ ions.

The analysis of the spectral properties in the wavelengths range of d-d transitions for the first time revealed preferable formation of tetrahedron Co^II^ chromophores versus the octahedral ones in the the Stöber nanoparticles, while the predominance of octahedral Co^II^ chromopheres was observed in the w/o nanoparticles. Both IR and Raman spectra indicate one more difference between the w/o and Stöber methods. The nanoparticles synthesized by the former method are decorated by the TX-100-based corona arisen from the TX-100 molecules adsorbed at the surface of the nanoparticles synthesized by the w/o method.

PXRD analysis of the synthesized nanoparticles indicated their amorphous nature, although he pracrystallinic inclusions into amorphous phase was revealed for the Stöber nanoparticles, which differentiate them from the w/o ones. The SAXS data revealed the heterogeneity of both nanoparticles, which is manifested by the presence of core zones with greater electron density than the shell zone. The sizes of the core zone are below those evaluated from the TEM images. The deviation between the sizes obtained by the different techniques for the w/o nanoparticles is no more than 20%. This enables to assume that the shell zone derives from the porous silica surface decorated by the TX-100-based corona. Both significant (above 50%) deviation revealed between the size values obtained by TEM and SAXS and specific PXRD profiles indicate the nano-structuring of the Stöber nanoparticles.

The electrochemical behavior of the Co^II^ ions within the hybrid nanoparticles indicates the insignificant influence of the synthetic procedure at the first oxidation and reduction steps, while the second Co^I^ to Co^0^ reduction step is greatly influenced by the synthetic procedure. In particular, the reversible Co^I^ to Co^0^ reduction is facilitated within the w/o nanoparticles. The electrochemical behavior of the hybrid nanoparticles provides an efficient basis for electrochemical analysis of glyphosate and HP(O)(OEt)_2_ with the lower detection limit at the level of 0.1 µmol·L^−1^ with the linearity of the electrochemical response in the concentration range from 0.1 to 80 µmol·L^−1^, short detection time, high stability, and easy operation. The sensing properties of the hybrid nanoparticles are also influenced by the synthetic method: the electrochemical response of the Stöber nanoparticles is able to discriminate glyphosate from HP(O)(OEt)_2_, while the w/o nanoparticles are more efficient in the sensing of the both toxicants without discrimination between them.

## Figures and Tables

**Table 1:** The elemental analysis data for C_30_H_24_B_2_CoF_8_N_6_.

Elements	C	H	B	Co	F	N
Anal. Calc., %	51.39	3.45	3.08	8.41	21.68	11.99
Found, %	51.41	3.41				

**Table 2:** Average diameters (d) in dried samples (TEM), Si:Co molar ratio (ICP-OES), average hydrodynamic diameters (D), polydispersity index (PDI) and zeta potential values (ζ) (DLS) in aqueous colloids of the differently synthesized Co^II^@SiO_2_ (C = 0.2 × g·L^−1^).

Synthetic Method, Precursor	d, nm	Si:Co Molar Ratio, %	D, nm	PDI	ζ, mV
Stöber, Co^II^(bpy)_3_	109 ± 20	100:0.243	225 ± 2	0.139	−51 ± 1
w/o, Co^II^(bpy)_3_	50 ± 5 ^1^	100:0.680	144 ± 1	0.114	−29 ± 1
Stöber, CoCl_2_	118 ± 10	100:0.130	154 ± 1	0.144	−51 ± 1
w/o, CoCl_2_	43 ± 3	100:0.578	118± 2	0.117	−38 ± 1

^1^ TEM image (Figure 1b) reveals an admixture of the small (2–5 nm) nanoparticles.

**Table 3:** The R_g_, R_g_*, D_max_, d_s_, FD, and V_part_ (averaged particle volume) values, the scattering intensity at *q* = 0 (I_0_, in arbitrary units), and the diameters of Co^II^@SiO_2_ and SiO_2_ nanoparticles synthesized by the both methods (d***^TEM^*** obtained by TEM method) are presented for the comparison.

Sample (Technique, Precursor)	R_g_, Å	R_g_*, Å	D_max_, nm	V_part_, Å^3^	I_0_, a.u.	d_s_, nm	FD	d^TEM^, nm
w/o, CoCl_2_	131.0 ± 6	130.9 ± 7	39.4 ± 0.6	122 × 10^5^	166000	33.8 ± 1.8	3.41	43 ± 3
w/o, Co^II^(bpy)_3_	145.5 ± 7	145.6 ± 5	43.4 ± 0.7	148 × 10^5^	229900	37.6 ± 1.3	3.29	50 ± 5
Stöber, CoCl_2_	164.6 ± 7	164.4 ± 8	50.1 ± 0.7	236 × 10^5^	84660	41.4 ± 2.1	2.61	118 ± 10
Stöber, Co^II^(bpy)_3_	139.4 ± 6	139.4 ± 7	43.7 ± 0.6	133 × 10^5^	60640	36.0 ± 1.8	2.69	109 ± 20
w/o, “empty”	128.0 ± 6	127.8 ± 7	36.4 ± 0.6	114 × 10^5^	183600	33.0 ± 1.8	3.53	48 ± 5
Stöber, “empty”	132.7 ± 8	132.6 ± 6	37.1 ± 0.6	101 × 10^5^	143200	34.0 ± 1.5	3.37	97 ± 5

**Table 4:** The potentials for Figure 7. Conditions: 25 °C, working electrode: CPE (graphite + ionic liquid + [Co particles]), Et_4_NBF_4_ background salt, H_2_O, 100 mV/s. Potentials vs. Fc^+^/Fc.

Sample	Reduction		Oxidation
	**^1^E_p_^c^/E_p_^a^_,_ V**	^2^E_p_^c^/E_p_^a^_,_ V	^1^E_p_^a^/E_p_^c^_,_ V
Stöber Co^II^@SiO_2_	−1.12/−0.90	−1.69/irrev	0.71/irrev
w/o Co^II^@SiO_2_	−1.15/−0.99	−1.38/−1.26	0.72/irrev

## Supplementary Materials

The following are available online at https://www.mdpi.com/2079-4991/10/7/1338/s1, Figure S1: UV-Vis spectra of supernatant diluted 300 times by ethanol after synthesis of w/o Co^II^@SiO_2_ (Co^II^(bpy)_3_ as precursor) (**blue**) and ethanol solutions of bpy (C = 5 × 10^−5^ mol·L^−1^, **red**) and Triton X-100 (C = 0.9 g·L^−1^, **black**), Figure S2: UV–Vis diffuse reflectance spectra of “empty” SiO_2_, Figure S3: SAXS diffraction intensity profiles at 23 °C (in logarithmic scale) top to bottom: (**blue**) w/o Co^II^@SiO_2_ (CoCl_2_ as precursor); (**red**) w/o Co^II^@SiO_2_ (Co^II^(bpy)_3_ as precursor); (**magenta**) Stöber Co^II^@SiO_2_ (CoCl_2_ as precursor); (**green**) Stöber Co^II^@SiO_2_ (Co^II^(bpy)_3_ as precursor); (**dark**) “empty” Stöber SiO_2_; (**dark blue**) “empty” w/o SiO_2_. Scattering vector s = 4πSinθ/λ, Å^−1^; λ = 1.5418Å is the wavelength of the X-ray beam, Figure S4: X-ray scattering curves of w/o CoII@SiO2 in logarithmic (left) and double logarithmic (right) scale. Different colors of curves correspond to eight consistent experiments, Figure S5: The fitting of experimental SAXS curve (points – experimental data, curves – simulation) and calculated distance distribution function p(r) for: (**a**) w/o Co^II^@SiO_2_ (CoCl_2_ as precursor); (**b**) w/o Co^II^@SiO_2_ (Co^II^(bpy)_3_ as precursor); (**c**) Stöber Co^II^@SiO_2_ (CoCl_2_ as precursor); (**d**) Stöber Co^II^@SiO_2_ (Co^II^(bpy)_3_ as precursor); (**e**) “empty” Stöber SiO_2_; (**f**) “empty” w/o SiO_2_, Figure S6: DPV voltammograms for w/o Co^II^@SiO_2_ (a) and Stöber Co^II^@SiO_2_ (b) in the absence and in the presence of increasing quantities of Glyphosate (from 0.1 µmol·L^−1^ to 80 µM: 0.1, 0.2, 0.3, 0.6, 1.2, 2.4, 5.0, 10.0, 20.0, 40.0, 80.0 µM). WE: CPE, H_2_O, 10^−1^ M Et_4_NBF_4_ Potentials vs. Ag/AgCl recalculated to Fc^+^/Fc. One-electron electron transfer during the oxidation and reduction of Co (II) is confirmed by the coincidence of the oxidation currents of ferrocene under the same conditions at the same analyte concentration (5 × 10^−3^ mol·L^−1^).

## References

[1] Antony R., Manickam S.T.D., Balakumar S. (2017). Cu(II), Co(II) and Ni(II) Complexes Installed on Functionalized Silica Surface for Hydrogen Peroxide Assisted Cyclohexane Oxidation. J. Inorg. Organomet. Polym. Mater..

[2] Vashurin A., Marfin Y., Tarasyuk I., Kuzmin I., Znoyko S., Goncharenko A., Rumyantsev E. (2018). Sulfonated *octa*-substituted Co(II) phthalocyanines immobilized on silica matrix as catalyst for Thiuram E synthesis. Appl. Organomet. Chem..

[3] Faraji A.R., Mosazadeh S., Ashouri F. (2017). Synthesis and characterization of cobalt-supported catalysts on modified magnetic nanoparticle: Green and highly efficient heterogeneous nanocatalyst for selective oxidation of ethylbenzene, cyclohexene and oximes with molecular oxygen. J. Colloid Interface Sci..

[4] Rajabi F., Luque R., Clark J.H., Karimi B., Macquarrie D.J. (2011). A silica supported cobalt (II) Salen complex as efficient and reusable catalyst for the selective aerobic oxidation of ethyl benzene derivatives. Catal. Commun..

[5] Chen L., Li B.-D., Xu Q.-X., Liu D.-B. (2013). A silica gel supported cobalt(II) Schiff base complex as efficient and recyclable heterogeneous catalyst for the selective aerobic oxidation of alkyl aromatics. Chin. Chem. Lett..

[6] Islam S.M., Ghosh K., Molla R.A., Roy A.S., Salam N., Iqubal M.A. (2014). Synthesis of a reusable polymer anchored cobalt(II) complex for the aerobic oxidation of alkyl aromatics and unsaturated organic compounds. J. Organomet. Chem..

[7] Marfin Y.S., Vashurin A.S., Rumyantsev E.V., Puhovskaya S.G. (2013). Sol–gel synthesis of highly effective catalyst based on cobalt tetrasulfophthalocyanine complex and silicon oxide. J. Sol Gel Sci. Technol..

[8] Ranaweera S.A., Rowe M.D., Walters K.B., Henry W.P., White M.G., Rodriguez J.M. (2017). Synthesis, characterization and catalytic activity of a cobalt catalyst: Silica-supported, bis(1,5-diphenyl-1,3,5-pentanetrionato)dicobalt(II)[Co_2_(dba)_2_]. Appl. Catal. A Gen..

[9] Li-Juan C., Fu-Ming M., Guang-Xing L. (2009). Co(II) Schiff base complexes on silica by sol–gel method as heterogeneous catalysts for oxidative carbonylation of aniline. Catal. Commun..

[10] Trujillano R., Lambert J.-F., Louis C. (2008). Chemistry of Silica-Supported Cobalt Catalysts Prepared by Cation Adsorption. 2. Neoformation of Cobalt Phyllosilicate. J. Phys. Chem. C.

[11] Yakubovich T.N., Ermokhina N.I., Bratushko Y.I., Zub Y.L., Chuiko A.A., Simindi L.I. (1991). Cobalt complex with 2.2′-bipyridyl immobilized on disperse silica surface in the reaction of p-hydroquinone oxidation. Comparison of the homogeneous and heterogeneous catalysis results. Dioxygen Activation and Homogeneous Catalytic Oxidation.

[12] Rautiainen A., Lindblad M., Backman L.B., Puurunen R.L. (2002). Preparation of silica-supported cobalt catalysts through chemisorption of cobalt(II) and cobalt(III) acetylacetonate. Phys. Chem. Chem. Phys..

[13] Yuanfa Y., Renxian Z., Shaofen Z., Qiaoling L., Xiaoming Z. (2003). Silica-supported poly-γ-aminopropylsilane Ni^2+^, Cu^2+^, Co^2+^ complexes: Efficient catalysts for Heck vinylation reaction. J. Mol. Catal. A Chem..

[14] Khrizanforov M.N., Fedorenko S.V., Strekalova S.O., Kholin K.V., Mustafina A.R., Zhilkin M.Y., Khrizanforova V.V., Osin Y.N., Salnikov V.V., Gryaznova T.V. (2016). A Ni(III) complex stabilized by silica nanoparticles as an efficient nanoheterogeneous catalyst for oxidative C–H fluoroalkylation. Dalton Trans..

[15] Khrizanforov M.N., Fedorenko S.V., Mustafina A.R., Khrizanforova V.V., Kholin K.V., Nizameev I.R., Gryaznova T.V., Grinenko V.V., Budnikova Y.H. (2019). Nano-architecture of silica nanoparticles as a tool to tune both electrochemical and catalytic behavior of Ni^II^@SiO_2_. RSC Adv..

[16] Budnikova Y., Bochkova O., Khrizanforov M., Nizameev I., Kholin K., Gryaznova T., Laskin A., Dudkina Y., Strekalova S., Fedorenko S. (2019). Selective C(sp^2^)-H Amination Catalyzed by High-Valent Cobalt(III)/(IV)-bpy Complex Immobilized on Silica Nanoparticles. ChemCatChem.

[17] Ryabchuk P., Agostini G., Pohl M.-M., Lund H., Agapova A., Junge H., Junge K., Beller M. (2018). Intermetallic nickel silicide nanocatalyst-A non-noble metal–based general hydrogenation catalyst. Sci. Adv..

[18] Dudkina Y.B., Gryaznova T.V., Osin Y.N., Salnikov V.V., Davydov N.A., Fedorenko S.V., Mustafina A.R., Vicic D.A., Sinyashin O.G., Budnikova Y.H. (2015). Nanoheterogeneous catalysis in electrochemically induced olefin perfluoroalkylation. Dalton Trans..

[19] Shabani-Nooshabadi M., Karimi-Maleh H., Tahernejad-Javazmi F. (2019). Fabrication of an electroanalytical sensor for determination of deoxyepinephrine in the presence of uric acid using CuFe_2_O_4_ nanoparticle/ionic liquid amplified sensor. J. Electrochem. Soc..

[20] Liu X. (2011). Electrochemical sensor for determination of parathion based on electropolymerization poly (safranine) film electrode. Int. J. Electrochem..

[21] Cao Y., Luona W., Chengyin W., Xiaoya H., Yunling L., Guoxiu W. (2019). Sensitive detection of glyphosate based on a Cu-BTC MOF/g-C_3_N_4_ nanosheet photoelectrochemical sensor. Electrochim. Acta.

[22] Shelkovnikov V.V., Novolokov K.Y. (2019). Voltammetric sensor for determining malathion and diazinon. Anal. Control.

[23] Tadayon F., Jahromi M.N. (2020). A sensitive and selective electrochemical determination of Diazinon based on Au–Pd/rGO–MWCNTs composite: Analytical application in water, fruit and vegetable samples. J. Iran. Chem. Soc..

[24] Song D., Li Y., Lu X., Sun M., Liu H., Yu G., Gao F. (2017). Palladium-copper nanowires-based biosensor for the ultrasensitive detection of organophosphate pesticides. Anal. Chim. Acta.

[25] Zahirifar F., Rahimnejad M., Abdulkareem R.A., Najafpour G. (2019). Determination of Diazinon in fruit samples using electrochemical sensor based on carbon nanotubes modified carbon paste electrode. Biocatal. Agric. Biotechnol..

[26] Mahmoudi E., Fakhri H., Hajian A., Afkhami A., Bagheri H. (2019). High-performance electrochemical enzyme sensor for organophosphate pesticide detection using modified metal-organic framework sensing platforms. Bioelectrochemistry.

[27] Sok V., Fragoso A. (2019). Amperometric biosensor for glyphosate based on the inhibition of tyrosinase conjugated to carbon nano-onions in a chitosan matrix on a screen-printed electrode. Microchim. Acta.

[28] Hua Q.T., Ruecha N., Hiruta Y., Citterio D. (2019). Disposable electrochemical biosensor based on surface-modified screen-printed electrodes for organophosphorus pesticide analysis. Anal. Methods.

[29] Hou W., Zhang Q., Dong H., Li F., Zhang Y., Guo Y., Sun X. (2019). Acetylcholinesterase biosensor modified with ATO/OMC for detecting organophosphorus Pesticides. New J. Chem..

[30] Tefera M., Admassie S., Tessema M., Mehretie S. (2015). Electrochemical sensor for determination of fenitrothion at multi-wall carbon nanotubes modified glassy carbon electrode. Anal. Bioanal. Chem. Res..

[31] Motaharian A., Motaharian F., Abnous K., Hosseini M.R.M., Hassanzadeh-Khayyat M. (2016). Molecularly imprinted polymer nanoparticles-based electrochemical sensor for determination of diazinon pesticide in well water and apple fruit samples. Anal. Bioanal. Chem..

[32] Budnikov G.K., Evtyugin G.A., Budnikova Y.G., Al′fonsov V.A. (2008). Chemically modified electrodes with amperometric response in enantioselective analysis. J. Anal. Chem..

[33] Khanmohammadi A., Ghazizadeh A.J., Hashemi P., Afkhami A., Arduini F., Bagheri H. (2020). An overview to electrochemical biosensors and sensors for the detection of environmental contaminants. J. Iran. Chem. Soc..

[34] Kokkinos C., Economou A. (2020). Recent advances in voltammetric, amperometric and ion-selective (bio)sensors fabricated by microengineering manufacturing approaches. Curr. Opin. Electrochem..

[35] Wu. L., Yan H., Wang J., Liu G., Xie W. (2019). Tyrosinase Incorporated with Au-Pt@SiO_2_ Nanospheres for Electrochemical Detection of Bisphenol, A. J. Electrochem. Soc..

[36] Jeerapan I., Poorahong S. (2020). Review-Flexible and Stretchable Electrochemical Sensing Systems: Materials, Energy Sources, and Integrations. J. Electrochem. Soc..

[37] Patel B.R., Noroozifar M., Kerman K. (2020). Review—Nanocomposite-Based Sensors for Voltammetric Detection of Hazardous Phenolic Pollutants in Water. J. Electrochem. Soc..

[38] Zhu N., Cai H., He P., Fang Y. (2003). Tris(2,2′-bipyridyl)cobalt(III)-doped silica nanoparticle DNA probe for the electrochemical detection of DNA hybridization. Anal. Chim. Acta.

[39] Zhang D., Wu Z., Xu J., Liang J., Li J., Yang W. (2010). Tuning the Emission Properties of Ru(phen)_3_^2+^ Doped Silica Nanoparticles by Changing the Addition Time of the Dye during the Stöber Process. Langmuir.

[40] Lian W., Litherland S.A., Badrane H., Tan W., Wu D., Baker H.V., Gulig P.A., Lim D.V., Jin S. (2004). Ultrasensitive detection of biomolecules with fluorescent dye-doped nanoparticles. Anal. Biochem..

[41] Ahkam Q.M., Khan E.U., Iqbal J., Murtaza A., Khan M.T. (2019). Synthesis and characterization of cobalt-doped SiO_2_ nanoparticles. Phys. B Condens. Matter.

[42] Bai S.H., Ogbourne S.M. (2016). Glyphosate: Environmental contamination, toxicity and potential risks to human health via food contamination. Env. Sci. Pollut. Res..

[43] Khrizanforov M., Gryaznova T., Sinyashin O., Budnikova Y. (2012). Aromatic perfluoroalkylation with metal complexes in electrocatalytic conditions. J. Organomet. Chem..

[44] Mustafina A.R., Fedorenko S.V., Konovalova O.D., Menshikova A.Y., Shevchenko N.N., Soloveva S.E., Konovalov A.I., Antipin I.S. (2009). Novel Highly Charged Silica-Coated Tb(III) Nanoparticles with Fluorescent Properties Sensitive to Ion Exchange and Energy Transfer Processes in Aqueous Dispersions. Langmuir.

[45] Khrizanforov M.N., Arkhipova D.M., Shekurov R.P., Gerasimova T.P., Ermolaev V.V., Islamov D.R., Miluykov V.A., Kataeva O.N., Khrizanforova V.V., Sinyashin O.G. (2015). Novel paste electrodes based on phosphonium salt room temperature ionic liquids for studying the redox properties of insoluble compounds. J. Solid State Electrochem..

[46] (2005). DIFFRAC Plus Evaluation package EVA.

[47] (2000). Small Angle X-ray Scattering.

[48] Konarev P.V., Volkov V.V., Sokolova A.V., Koch M.H.J., Svergun D.I. (2003). PRIMUS: A Windows PC-based system for small-angle scattering data analysis. J. Appl. Cryst..

[49] Arriagada F.J., Osseo-Asare K. (1999). Controlled hydrolysis of tetraethoxysilane in a nonionic water-in-oil microemulsion: A statistical model of silica nucleation. Colloids Surf. A Physicochem. Eng. Asp..

[50] Liver E. (1987). Elektronnaya Spektroskopiya Neorganicheskih Soedineniy (Electron Spectroscopy of Inorganic Compounds).

[51] Alexandru M.-G., Velickovic M., Hrubaru K.M.-M., Draghici C. (2013). Two complexes of Co(II) and Pd(II) formed in reaction with a mono-T.C. oxazoline derivative. Spectroscopic characterization and cytotoxic evaluation. J. Mol. Struct..

[52] Verberckmoes A.A., Weckhuysen B.M., Schoonheydt R.A. (1998). Spectroscopy and coordination chemistry of cobalt in molecular sieves. Microporous Mesoporous Mater..

[53] Gerasimova T., Shekurov R., Gilmanova L., Laskin A., Katsyuba S., Kovalenko V., Khrizanforov M., Milyukov V., Sinyashin O. (2018). IR and UV study of reversible water-induced structural transformations of poly(manganese 1,10 -ferrocenediyl-bis(Hphosphinate)) and poly(cobalt 1,10-ferrocenediyl-bis(H-phosphinate)). J. Mol. Struct..

[54] Shekurov R., Khrizanforova V., Gilmanova L., Khrizanforov M., Miluykov V., Kataeva O., Yamaleeva Z., Burganov T., Gerasimova T., Khamatgalimov A. (2019). Zn and Co redox active coordination polymers as efficient electrocatalysts. Dalton Trans..

[55] Yousef T.A., El-Reash G.M.A., El-Gammal O.A., Bedier R.A. (2012). Co(II), Cu(II), Cd(II), Fe(III) and U(VI) complexes containing a NSNO donor ligand: Synthesis, characterization, optical band gap, in vitro antimicrobial and DNA cleavage studies. J. Mol. Struct..

[56] Kang W., Spanjers C.S., Rioux R.M., Hoefelmeyer J.D. (2013). Synthesis of *brookite* TiO_2_nanorods with isolated Co(II) surface sites and photocatalytic degradation of 5,8-dihydroxy-1,4-naphthoquinone dye. J. Mater. Chem. A.

[57] Islam S., Bakhtiar H., Aziz M.S.B.A., Duralima M.B., Riaz S., Naseemb S., Abdullha M.B., Osman S.S. (2018). CR incorporation in mesoporous silica matrix for fiber optic pH sensing. Sens. Actuators A Phys..

[58] Islam S., Bakhtiar H., Naseem S., Aziz M.S.B.A., Biden N., Riaz S., Ali J. (2018). Surface functionality and optical properties impact of phenol red dye on mesoporous silica matrix for fiber optic pH sensing. Sens. Actuators A Phys..

[59] Chen C., Xu J., Zhang Q., Ma H., Miao H., Zhou L. (2009). Direct synthesis of bifunctionalized hexagonal mesoporous silicas and its catalytic performance for aerobic oxidation of cyclohexane. J. Phys. Chem. C.

[60] Fouad O.A., Makhlouf S.A., Ali G.A.M., El-Sayed A.Y. (2011). Cobalt/silica nanocomposite via thermal calcination-reduction of gel precursors. Mater. Chem. Phys..

[61] Genovese D., Rampazzo E., Bonacchi S., Montalti M., Zaccheroni N., Prodi L. (2014). Energy transfer processes in dye-doped nanostructures yield cooperative and versatile fluorescent probes. Nanoscale.

[62] Koirala R., Safonova O.V., Pratsinis S.E., Baiker A. (2018). Effect of cobalt loading on structure and catalytic behavior of CoO_x_/SiO_2_ in CO_2_-assisted dehydrogenation of ethane. Appl. Catal. A.

[63] Okamoto Y., Nagata K., Adachi T., Imanaka T., Inamura K., Takyu T. (1991). Preparation and Characterization of Highly Dispersed Cobalt Oxide and Sulfide Catalysts Supported on SiO_2_. J. Phys. Chem..

[64] Masalov V.M., Kudrenko E.A., Grigoryeva N.A., Ezdakova K.V., Roddatis V.V., Sukhinina N.S., Arefev M.V., Mistonov A.A., Grigoriev S.V., Emelchenko G.A. (2013). Direct observation of the shell-like structure of SiO_2_ partices synthesized by the multistage Stober method. Nano Brief Rep. Rev..

[65] Fouilloux S., Désert A., Taché O., Spalla O., Daillant J., Thill A. (2010). SAXS exploration of the synthesis of ultra monodisperse silica nanoparticles and quantitative nucleation growth modeling. J. Colloid Interface Sci..

[66] Fouilloux S., Daillant J., Thill A. (2012). Single step synthesis of 5–30 nm monodisperse silica nanoparticles: Important experimental parameters and modeling. Colloids Surf. A Physicochem. Eng. Asp..

[67] De Almeida L.K.S., Chigome S., Torto N., Frost C.L., Pletschke B.I. (2015). A novel colorimetric sensor strip for the detection of glyphosate in water. Sens. Actuators B Chem..

