# Supplementary material for: Synthetic Tuning of CoII-Doped Silica Nanoarchitecture Towards Electrochemical Sensing Ability
Source: Nanomaterials (Basel). 2020 Jul 9;10(7):1338. doi: 10.3390/nano10071338 (PMC7407651; doi:10.3390/nano10071338)
Supplement: Supplementary file 1 [file nanomaterials-10-01338-s001.pdf]

Supplementary materials

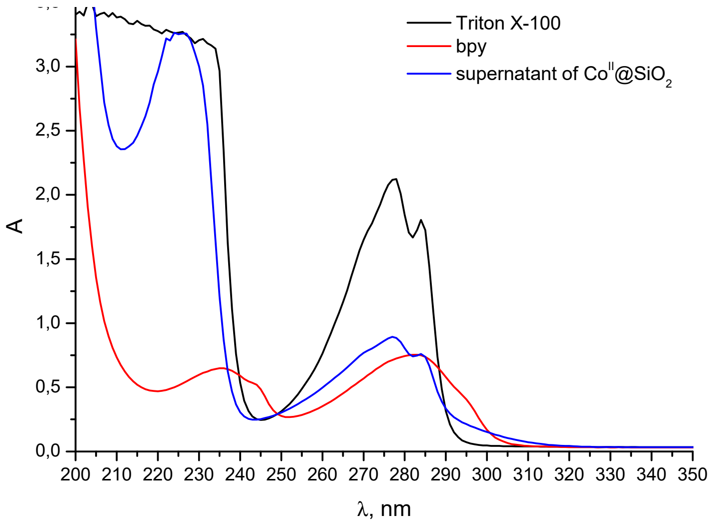

Figure 1. UV-vis spectra of supernatant diluted 300 times by ethanol after synthesis of w/o $Co^{II}@SiO_2$ ($Co^{II}(bpy)_3$ as precursor) (**blue**) and ethanol solutions of bpy ($C = 5\times10^{-5}$ mol·L^{-1}, **red**) and Triton X-100 ($C = 0.9$ g·L^{-1}, **black**).

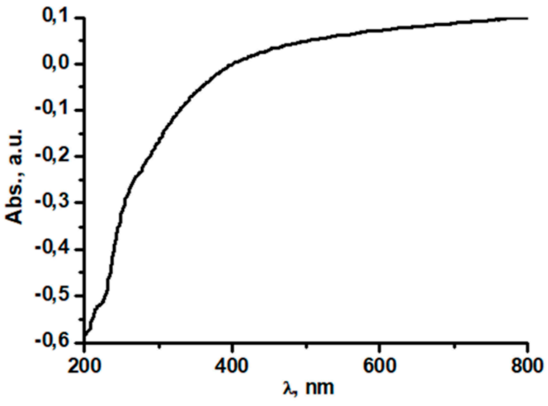

Figure S2. UV–Vis diffuse reflectance spectra of "empty" SiO_2.

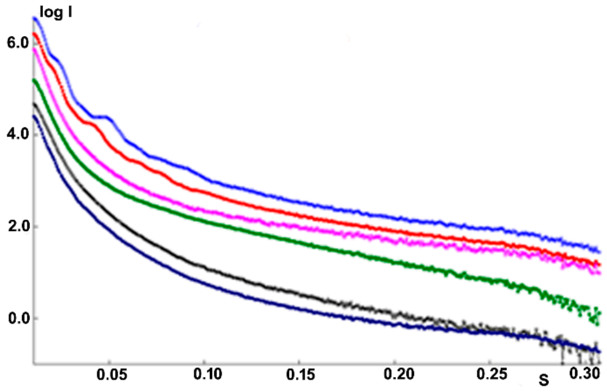

Figure S3. SAXS diffraction intensity profiles at 23 °C (in logarithmic scale) top to bottom: **(blue)** w/o $Co^{II}@SiO_2$ ($CoCl_2$ as precursor); **(red)** w/o $Co^{II}@SiO_2$ ($Co^{II}(bpy)_3$ as precursor); **(magenta)** Stöber $Co^{II}@SiO_2$ ($CoCl_2$ as precursor); **(green)** Stöber $Co^{II}@SiO_2$ ($Co^{II}(bpy)_3$ as precursor); **(dark)** "empty" Stöber SiO_2; **(dark blue)** "empty" w/o SiO_2. Scattering vector $s = 4\pi Sin\theta/\lambda$, Å^{-1}; $\lambda = 1.5418$Å is the wavelength of the X-ray beam.

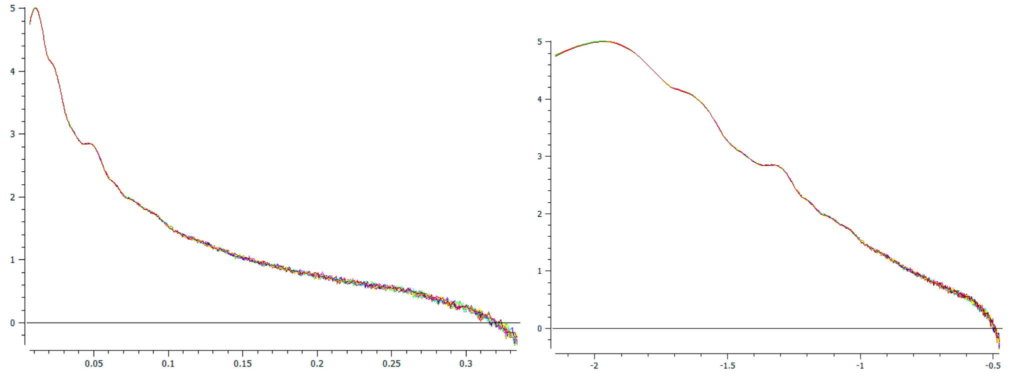

Figure S4. X-ray scattering curves of w/o $Co^{II}@SiO_2$ in logarithmic (**left**) and double logarithmic (**right**) scale. Different colors of curves are corresponded to 8 consistent experiments.

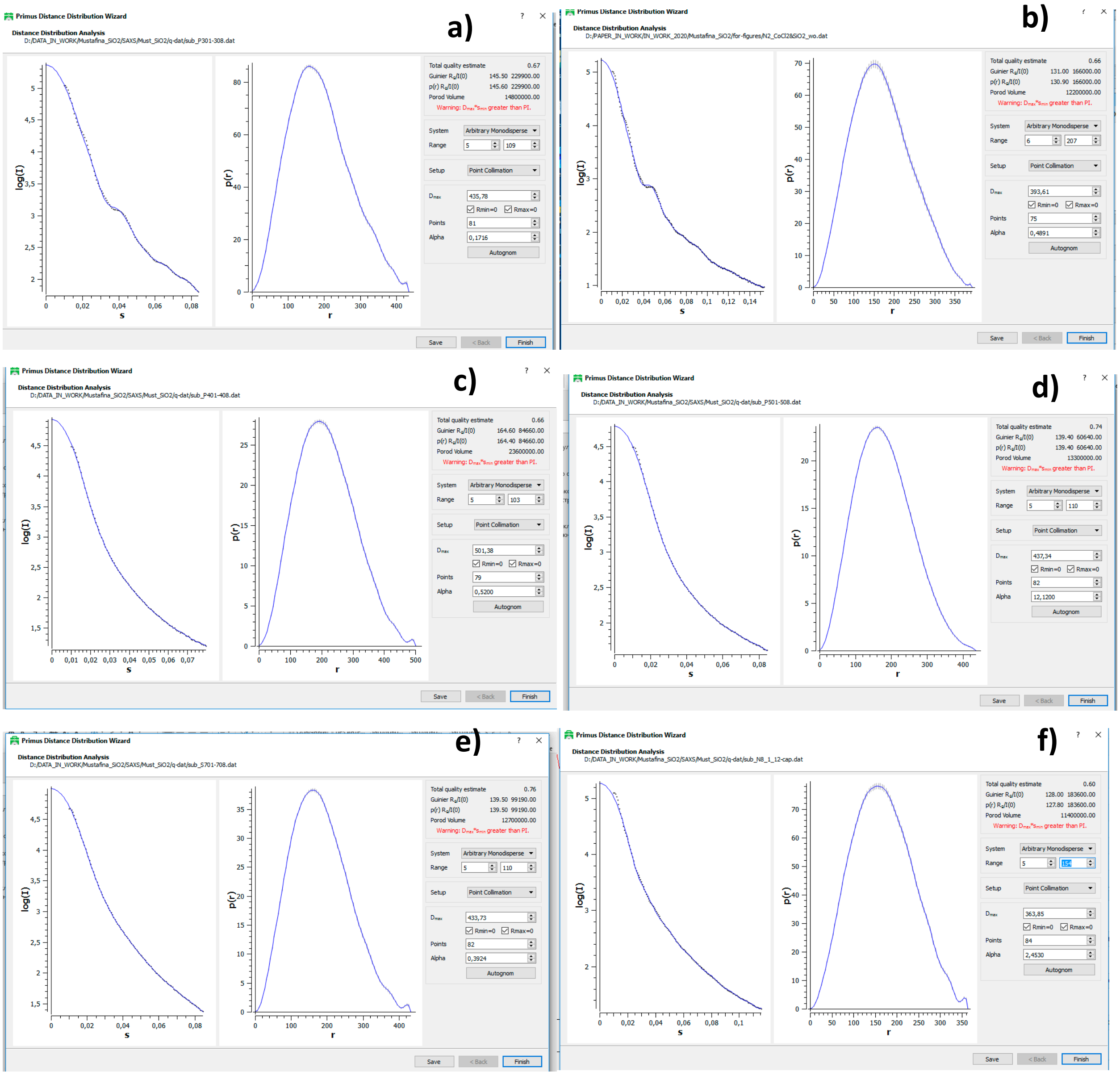

Figure S5. The fitting of experimental SAXS curve (points – experimental data, curves – simulation) and calculated distance distribution function p(r) for: (**a**) w/o $Co^{II}@SiO_2$ ($CoCl_2$ as precursor); (**b**) w/o $Co^{II}@SiO_2$ ($Co^{II}(bpy)_3$ as precursor); **c)** Stöber $Co^{II}@SiO_2$ ($CoCl_2$ as precursor); (**d**) Stöber $Co^{II}@SiO_2$ ($Co^{II}(bpy)_3$ as precursor); (**e**) "empty" Stöber SiO_2; (**f**) "empty" w/o SiO_2.

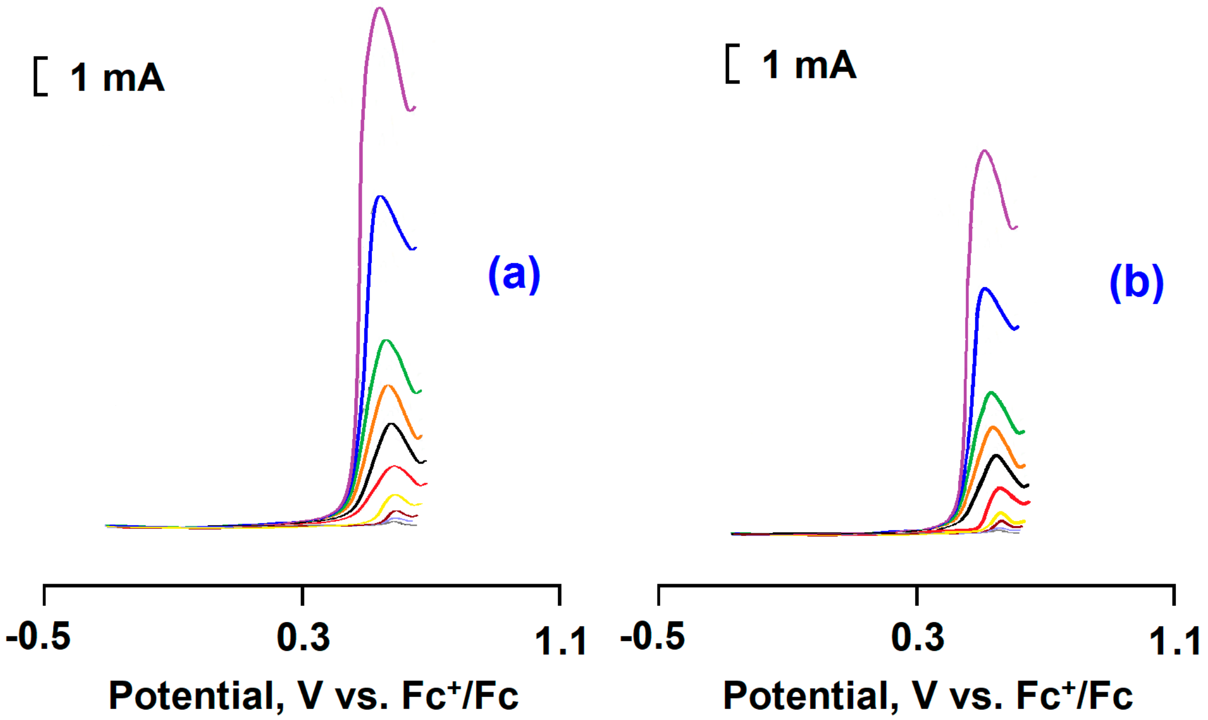

Figure S6. DPV voltammograms for w/o Co^{II}@SiO_2 (**a**) and Stöber Co^{II}@SiO_2 (**b**) in the absence and in the presence of increasing quantities of Glyphosate (from 0.1 to 80 μmol·L^{-1}: 0.1, 0.2, 0.3, 0.6, 1.2, 2.4, 5.0, 10.0, 20.0, 40.0, 80.0 μM). WE: CPE, H_2O, 10^{-1} M Et_4NBF_4 Potentials vs. Ag/AgCl recalculated to Fc^+/Fc. One-electron electron transfer during the oxidation and reduction of Co (II) is confirmed by the coincidence of the oxidation currents of ferrocene under the same conditions at the same analyte concentration (5×10^{-3} mol·L^{-1}).

The area of the electrode was calculated by the Randles-Sevcik equation:

$$ip = (2.69\cdot10^5) \times n^{3/2} \times A\ D^{1/2} \times C \times v^{1/2} \quad (1)$$

where n is the number of moles of electrons transferred in the reaction, ip is peak current, A is the area of the electrode, C is the analyte concentration (in moles/cm^3), D is the diffusion coefficient, and v is the scan rate of the applied potential. A = 2.0008 mm^2
